# A Biomimetic, SoC-Based Neural Stimulator for Novel Arbitrary-Waveform Stimulation Protocols

**DOI:** 10.3389/fnins.2021.697731

**Published:** 2021-07-29

**Authors:** Stanislav Culaclii, Po-Min Wang, Giuliano Taccola, William Yang, Brett Bailey, Yan-Peng Chen, Yi-Kai Lo, Wentai Liu

**Affiliations:** ^1^Department of Bioengineering, University of California, Los Angeles, Los Angeles, CA, United States; ^2^Jet Propulsion Laboratory, California Institute of Technology, Pasadena, CA, United States; ^3^Neuroscience Department, International School for Advanced Studies, Trieste, Italy; ^4^Department of Integrative Biology and Physiology, University of California, Los Angeles, Los Angeles, CA, United States; ^5^Department of Electrical Engineering, University of California, Los Angeles, Los Angeles, CA, United States; ^6^Niche Biomedical Inc., Los Angeles, CA, United States; ^7^California NanoSystems Institute, University of California, Los Angeles, Los Angeles, CA, United States; ^8^Brain Research Institute, University of California, Los Angeles, Los Angeles, CA, United States

**Keywords:** neural stimulator, biomimetic, SoC, multi-channel, arbitrary waveform, implant, wireless, control logic

## Abstract

Novel neural stimulation protocols mimicking biological signals and patterns have demonstrated significant advantages as compared to traditional protocols based on uniform periodic square pulses. At the same time, the treatments for neural disorders which employ such protocols require the stimulator to be integrated into miniaturized wearable devices or implantable neural prostheses. Unfortunately, most miniaturized stimulator designs show none or very limited ability to deliver biomimetic protocols due to the architecture of their control logic, which generates the waveform. Most such designs are integrated into a single System-on-Chip (SoC) for the size reduction and the option to implement them as neural implants. But their on-chip stimulation controllers are fixed and limited in memory and computing power, preventing them from accommodating the amplitude and timing variances, and the waveform data parameters necessary to output biomimetic stimulation. To that end, a new stimulator architecture is proposed, which distributes the control logic over three component tiers – software, microcontroller firmware and digital circuits of the SoC, which is compatible with existing and future biomimetic protocols and with integration into implantable neural prosthetics. A portable prototype with the proposed architecture is designed and demonstrated in a bench-top test with various known biomimetic output waveforms. The prototype is also tested *in vivo* to deliver a complex, continuous biomimetic stimulation to a rat model of a spinal-cord injury. By delivering this unique biomimetic stimulation, the device is shown to successfully reestablish the connectivity of the spinal cord post-injury and thus restore motor outputs in the rat model.

## Introduction

**FIGURE 1:**
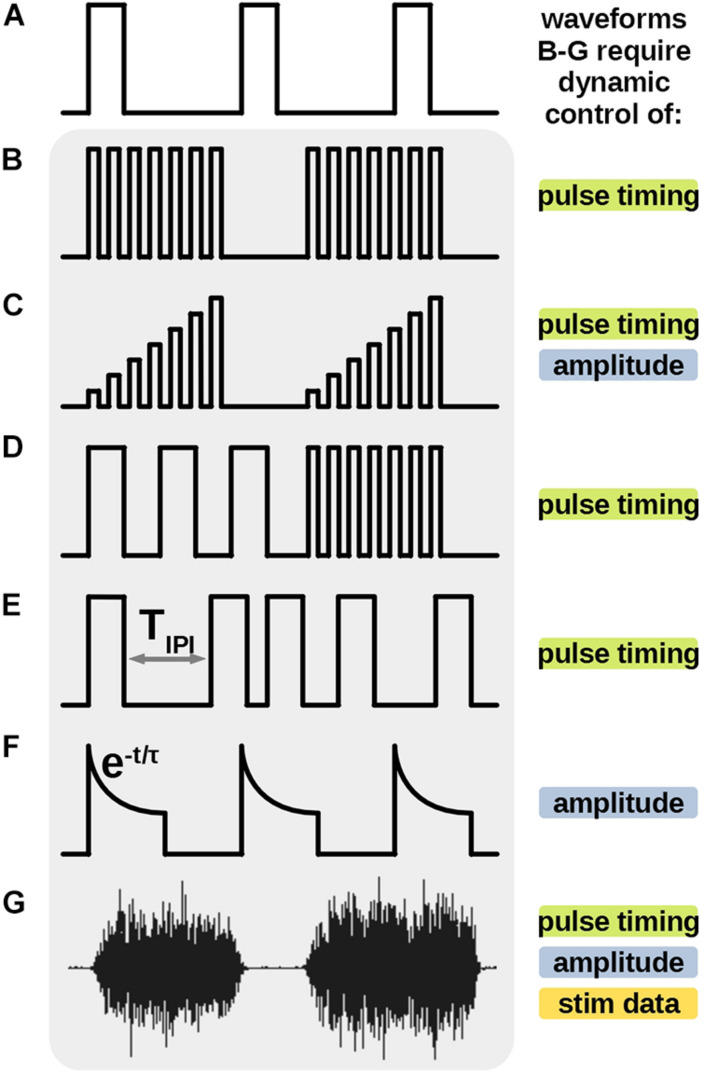
Previously demonstrated novel protocols, uniquely different from the traditional uniform pulse train **(A)** require additional stimulator features to be generated. High frequency bursts **(B,C)** pulse frequency modulation (FM) **(D)** varying inter-pulse-interval **(E)** and the biomimetic waveform (G) require dynamic control of timing of individual stimulus pulses. Amplitude ramp **(C)** energy efficient stimulus **(F)** and biomimetic waveform **(G)** also require dynamic amplitude during the stimulus output. Uniquely, the biomimetic waveform **(G)** requires an ability to store, process and output a relatively large amount of stimulus data points to recreate the protocol. Some, but not all such features were demonstrated in previous works. All these features are available in the proposed stimulator architecture.

Electrical stimulation has been long employed as a treatment for neural disorders, such as Parkinson’s (Benabid et al., 1991), epilepsy (Mogul and van Drongelen, 2014); treatment for pain (Gerasimenko et al., 2008); restoration of sensory disorders, such as vision (Weiland et al., 2005) and hearing (Moore and Shannon, 2009); rehabilitation of locomotion for spinal cord injury patients (Taccola et al., 2018), among others. In most applications the stimulation treatment is delivered in the form of charge-controlled, constant-current or voltage pulses, usually repeating as uniform pulse trains (Merrill et al., 2005). Accordingly, most novel neural-prosthetic stimulator designs focus on this type of conventional stimulation waveforms. Such stimulator designs, as in work (Stanslaski et al., 2012), fundamentally employ circuits with constant current or voltage sources and timers to turn the sources on and off with a predefined uniform frequency (Figures 1A,B).

Novel stimulation waveforms, shaped unlike the uniform pulse trains, have been shown to be advantageous for specific applications in therapeutics and research. Neural prosthetics require unique design architectures to accommodate such waveforms (Figure 1). For example, amplitude ramps, which require dynamic control of amplitude in each stimulation pulse (Figure 1C), have been shown to induce asynchronous neural firings in the targeted tissue, leading to a more natural neural activity (Formento et al., 2020). Neural stimulator employed by Wagner et al. (2018) produces stimulation trains with multiple discrete frequencies (Figure 1D) to enable locomotion in patients with a spinal cord injury. Brocker et al. (2013), Eles et al. (2020), and Soto-Breceda et al. (2018) demonstrate delivery of a series of pulses with irregular inter-pulse-intervals to reduce synaptic fatigue in retina tissue or improve therapeutic effects in Parkinson’s treatments. Stimulators designed for these protocols require a dynamic control of timing of each individual pulse in the series (Figure 1E). Energy efficient waveforms with exponential decaying amplitudes (Figure 1F) are demonstrated by Lee et al. (2018), and require additional circuitry to control the amplitude of the current pulse during its output. Finally, Figure 1G shows a novel biomimetic stimulation waveform from work by Taccola et al. (2020a, b) which was titled Dynamic Stimulation (DS) and was created from an electromyography (EMG) recording of the soleus (Sol) muscle of a healthy rat during stepping. The complex waveform is empirically shown to be significantly more effective than pulse trains in increasing the excitability and neural connectivity of the spinal cord, potentially advantageous in therapy post spinal cord injury. This protocol requires a stimulator architecture which can dynamically control all above parameters of the output current and reproduce a relatively long, continuous, arbitrary waveform data, posing the most complex challenge for stimulator design.

At the same time, long-term treatments using the above stimulation protocols often require implementation of the stimulator as an implantable neuroprosthetic, prompting integrated and miniaturized design in the form of a System-on-Chip (SoC) (Stanslaski et al., 2012; Sun and Morrell, 2014; Yip et al., 2015). Although commercial stimulators, such as STG 4008 (Multi Channel Systems, Reutlingen, Germany) used by Taccola et al. (2020a, b) support continuous arbitrary waveforms, they cannot be translated to miniaturized implantable neural prosthetics. Several state-of-the-art SoC-based designs, targeting implantable applications, have been shown to support arbitrary stimulation waveforms (Noorsal et al., 2012; Yip et al., 2015; Kassiri et al., 2017; Piech et al., 2020). Yet, designs (Noorsal et al., 2012; Yip et al., 2015; Kassiri et al., 2017) allow for maximum length of the arbitrary waveform of 8–64 points, limiting the application to controlling the shape of short repeating pulses, rather than stimulating with true complex biomimetic signals. Architecture of the design in Piech et al. (2020) allows for a longer stream of arbitrary parameters for the output stimulus, but limits the output signal’s duty cycle to <50% at amplitude resolution of 3-bits, due to the inherent power limitations of the design, in which a millimeter-sized neural implant is remotely powered by ultrasound waves. This restricts the output to a series of discrete stimulus pulses rather than a continuous biomimetic waveform.

A novel stimulator system is proposed to address the need for supporting existing and future novel arbitrary biomimetic waveforms, as well as all other waveforms outlined above, while also being suitable for implantable applications. The proposed 3-tier control architecture, described in section “System Architecture”, distributes the waveform generating logic onto SoC, adopted from prior work (Lo et al., 2016), and the external components of the system. Empowered by an additional firmware layer, it provides flexibility to support a multitude of stimulation protocols, while being compatible with implantable neural prosthetics. A prototype is built and its performance is demonstrated in section “Bench-Top Test” at the benchtop level. Finally, the prototype is tested *in vivo* to increase excitability of the spinal cord and restore connectivity in animal subjects with spinal cord injury, and the results are discussed in section “*In vivo* Tests”. This work expands on our previous report (Wang et al., 2019), by adding further design details, signal analysis, new bench-top test results and *in vivo* animal testing.

## Materials and Methods

### Quantifying the Biomimetic Stimulator’s Performance

The main requirement for the proposed system is to preserve the key desired features of the biomimetic stimulation protocol. These features are derived and quantified by analysis of the biomimetic signal in the section below. Additional electrical performance considerations, related to the data link and the analog circuitries, are discussed in a further section. The theoretical values and the values measured from the prototyped system are summarized Table 1.

**TABLE 1:** Performance of the biomimetic stimulator prototype.

System’s Signal Analysis: Theoretical vs. Measured				

Parameter	Original DS signal	Time-cropped and quantized signal	Measured system output	Δ (Original DS – measured output)
Amplitude range of positive peaks	181 μA/0.344 V^4^	176 μA/0.334 V^4^	0.376 V	+9.3%^1^
Amplitude range of negative peaks	225 μA/0.428 V^4^	223 μA/0.424 V^4^	0.418 V	−2.3%^1^
Wiener entropy	−2.19 dB	−2.17 dB	−1.87 dB	+3.8%(+0.32 dB)^1^

**System’s electrical performance**				

Parameter	Stimulation data bitrate	Output compliance voltage^3^	Maximum accepted electrode impedance^3^	Charge balance mechanism^3^
Value	133 kbps	± 10 V	24 kΩ+^2^	Passive charge dissipation

*^1^Values include 60 Hz noise and a high-frequency noise aliased into the signal band during the oscilloscope data capture. ^2^Upper impedance limit is stated for maximum stimulation current of 500 μA; if current is lowered, the limit increases. ^3^Values presented for reference from prior work (Lo et al., 2017). ^4^Current values of the signal are converted to voltage through R_S_ resistor of the Randles Cell model.*

#### Signal Analysis of the Biomimetic Protocol

**FIGURE 2:**
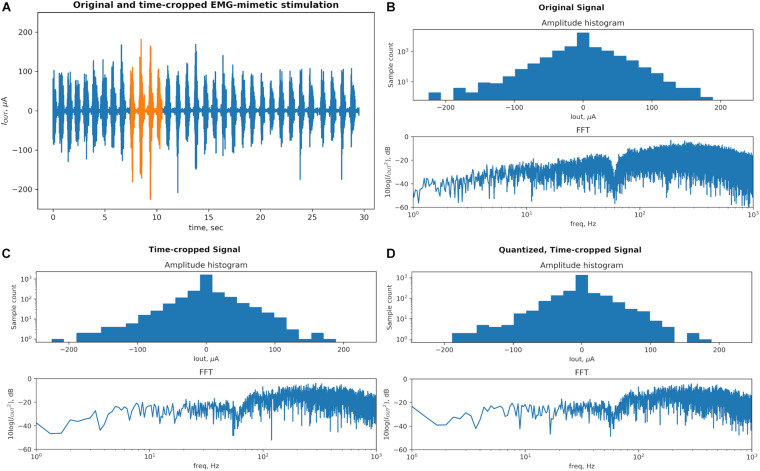
A physiological recording used as a biomimetic stimulation protocol Dynamic Stimulation (DS) is transformed to conform to the typical hardware limitations of the System-on-Chip (SoC)-based stimulators. The key characteristics responsible for therapeutic efficacy of this signal (amplitude and frequency modulations) are preserved through the transformation process. **(A)** Original signal (blue) contains bursts corresponding to rhythmic stepping of an animal on a treadmill. The signal was strategically time-cropped, from 30 to 3 s, to preserve the wide range of peak amplitudes and frequencies while saving memory space in the proposed stimulator system. **(B)** Original signal’s statistic of peak amplitudes and frequency content defines a baseline for the comparison. The dip in the spectrum at 60 Hz is created by the “line” noise filter. **(C)** The time-cropped signal’s peak amplitude counts and the number of FFT samples are reduced proportionally with the length. The general shape of the histogram and frequency content is notably generally unchanged. A dip at the ∼140 μA bin results from a reduced number of high amplitude peaks in the time-cropped signal as it contains less high amplitude bursts as compared to the original signal. **(D)** The quantization step reduces the resolution from the 14-bit precision of the recording amplifier that captured the original signal to the 8-bit output resolution of the neural stimulator SoC employed in this work. The amplitude statistics are and FFT are notably unchanged and this final signal is used in the proposed system and the *in vivo* testing.

The novel biomimetic waveform, DS (Figure 1G) is used in the *in vivo* test of our proposed stimulator prototype. DS contains two key features – amplitude modulation (AM) and frequency modulation (FM) – which are thought to contribute to its unique efficacy in recruitment of neural networks. A biomimetic stimulator design thus needs to preserve such key modulation characteristics in its output. However, a high-resolution stimulation output requires a hardware design that has a larger size and a higher power consumption, which conflicts with the design requirements of implantable or wearable applications. The miniaturization of critical stimulator components as SoCs results in decreased resolution in timing and output current and reduced memory sizes, which may distort the output stimulus signal and change its key AM and FM modulations, as compared to desktop-sized, non-portable commercial neural stimulators. In order to evaluate the impact of reduced resolution on the key AM and FM features, the DS signals are analyzed (blue trace in Figure 2A). The signal is linearly scaled to limit the amplitude peaks to ±225 μA (450 μA peak-to-peak), which was previously found to be therapeutically effective in rat-model experiments similar to the *in vivo* testing in section “*In vivo* Tests”. Next, the signal is cropped in time domain from 30 to 3 s, retaining the original 2 ksps sampling rate. The chosen length of the crop at the original sampling rate preserves the frequency variation of the signal. The location of the crop is strategically selected to retain the large amplitude variations of complete EMG bursts (orange trace in Figure 2A).

To evaluate effects of the DS signal’s transformation on its key features we plot a histogram of peak amplitudes and the fast fourier transform (FFT) of the signal at each step, which are displayed in Figure 2. A peak is defined as an extremum (positive or negative) between each pair of consecutive zero-crossings. Figure 2B shows that the original signal contains peaks with amplitude values spanning from signal’s minimum to maximum. The high count around the 0-amplitude represents noise in the recorded physiological signal. The signal’s distinct high-amplitude EMG peaks represent the motoneuron outputs and are fewer compared to low-amplitude peak activity. The histogram’s *y*-axis is plotted on a log scale to increase visibility of both high-count and low-count bins. The FFT spectrum is plotted on a 10-log dB scale after the signal is squared to represent its power. The FFT spectrum shows a notch at 60 Hz created by a line noise filter and a relatively higher power in the 100–1000 Hz band as typical of EMG signals.

Figure 2C shows that cropping the signal in time domain by 10× reduces the values on the bin counts in the histogram proportionally, as expected, but the distribution of the peak amplitudes remains the same. The number of points of the FFT plot is reduced proportionally to the signal length but the frequency content is otherwise unchanged. The quantization step shown in Figure 2D transfers some peak amplitudes across histogram bins but otherwise no notable difference is seen.

In addition to qualitative observations of the histograms, the modulation of the amplitude is also quantified by the range between the largest and the smallest amplitude peaks, measured separately in positive and negative directions. The peak amplitude ranges are 181 and 225 μA for the original DS signal (positive and negative, respectively), and 176 and 223 μA for the pre-processed signal.

The modulation in frequency is quantified by the flatness of the frequency spectrum, which indicates how uniformly the signal power is distributed over its bandwidth. Wiener entropy defines spectral flatness as the ratio of geometrical mean to arithmetic mean of the power in all frequency bins, usually reported in decibels.

(1) $$\begin{array}{c} Wienerentropy=\frac{\sqrt[{N}]{\prod_{n=0}^{N-1}x\left(n\right)}}{\frac{\sum_{n=0}^{N-1}x\left(n\right)}{N}}=\frac{\text{exp}\left(\frac{1}{N}\sum_{n=0}^{N-1}\ln x\left(n\right)\right)}{\frac{1}{N}\sum_{n=0}^{N-1}x\left(n\right)},\\ N=numberoffrequencybins \end{array}$$

This measurement will thus be much below 0 dB for a signal with a frequency content concentrated in single or few frequency bands, as is the case for traditional tonic stimulation protocols. The biomimetic protocol should instead measure close to 0 dB, signifying that the frequencies are modulated more evenly across the spectrum. Indeed, the Wiener entropy for the original DS signal and the pre-processed signal are −2.19 and −2.17 dB, respectively. These quantities are recorded are recorded in Table 1.

The preliminary signal analysis suggests that the key AM and FM features of the DS waveform can be retained while it is carefully condensed to reduce the resource usage in the proposed stimulator. To ensure that the prototyped stimulator can deliver this signal correctly, its output is measured in section “Bench-Top Test” at bench-top test and compared to the quantities above, and its efficacy is empirically validated *in vivo* in section “*In vivo* Tests.”

#### Electrical Performance of the Biomimetic Stimulator

A key requirement for the implementation of the proposed architecture is to adhere to the maximum bitrate available for its data link. Compared to the conventional, tonic stimulation, the biomimetic protocol contains more information due to its longer length and varying amplitude, which needs to be transmitted to the stimulating component (here: the SoC). In the case of a wireless implementation, the available bitrate depends on the data telemetry link employed. Prior art on neural implantable interfaces by Noorsal et al. (2012) and Piech et al. (2020) report transmission bitrates of 2 and 1.85 Mbps, respectively. Additionally, a commercially available medical-band RF transceiver chip ZL70103 (Microchip, Chandler, AZ, United States) can support 800 ksps raw wireless data rate distributed as 70% effective data and 30% communication overhead. Finally, the SoC adopted here includes an on-chip telemetry with a previously demonstrated continuous transmission rate of 2 Mbps, free of overhead aside from what is already included into the native stimulation data packets. The actual command bit rate to the SoC during output of biomimetic protocol is measured in section “Bench-Top Test” and recorded in Table 1, to confirm compatibility with reported maximum data rates.

Other commonly cited analog electrical performance measurements of the stimulator, such as output compliance voltage, accepted electrode impedance range, charge cancelation mechanisms, are independent of the proposed control architecture, and are instead specific to the *in vivo* applications targeted by the stimulator. These properties of the adopted stimulator SoC were measured in prior works for application to spinal cord implants and are summarized in Table 1 for reference.

### System Architecture

Neural stimulators take stimulation parameters as an input and administer the stimulation by repeatedly turning its output current or voltage sources on and off at designated times according to control logic. Specifically, traditional implementations of a miniaturized or implantable neural interface typically achieve this by employing an on-chip controller designed to deliver a specific type of stimulation. An external device communicating with the stimulator then only serves the purpose of updating a limited set of stimulator’s parameters and triggering a start and stop of a predefined protocol (Stanslaski et al., 2012; Shahdoost et al., 2018; Zhou et al., 2019). Such designs have no or limited abilities to support the next-generation biomimetic protocols discussed in sections “Introduction” and “Quantifying the Biomimetic Stimulator’s Performance.” If the physiological research evolves to require a significant update to the *in vivo* experiment design, a need to change the default mode of stimulation can arise which can require a difficult hardware revision.

For example, Wagner et al. (2018) collaborated with Medtronic (Minneapolis, MN, United States) to upgrade the hardware of a single-frequency periodic stimulator Activa RC (Medtronic, Minneapolis, MN, United States) to enable support for multiple discrete frequencies as needed during the experiments. Any future changes to experiment requirements, such as a need to modulate amplitude, phase and/or frequency across an arbitrary continuous range, would necessitate further hardware changes, which are especially expensive in implantable systems. This expense can be avoided in a design where the controller of the stimulation waveform is partially distributed outside of the neural interface itself, where the changes can be easily made by firmware and software updates.

To this end, the stimulator architecture proposed here provides the flexibility to support both conventional pulse-train protocols and other existing and future biomimetic protocols by integrating software, firmware, as well as digital circuitry into a unified control logic.

#### Logic Architecture for Arbitrary Waveform Generation

**FIGURE 3:**
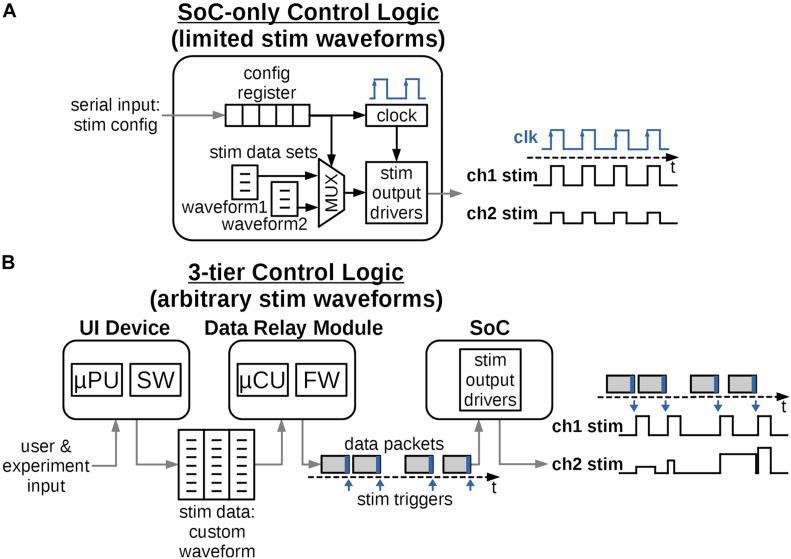
Possible ways to distribute control logic to create the required stimulation waveforms. **(A)** The logic is integrated solely into the digital circuits of the SoC. Provisions are made for a limited set of output waveforms with short, periodically repeating patterns. The design is fixed and future waveform protocols would require challenging and costly redesign of the SoC. **(B)** The control logic is distributed into 3-tiers and accommodates a variety of arbitrary and biomimetic stimulation waveforms. The logic can be reprogrammed for future protocols relatively easily and at low cost.

Figure 3 shows options for an architecture of a stimulator’s control logic. A traditional approach in Figure 3A uses on-chip digital circuits to form a memory register and stimulation-specific finite-state machines to generate the output signal. The memory register receives basic configuration parameters from an external device, and the state machine executes “hardwired,” fixed stimulation patterns, which are typically periodic square waves with constant frequency, amplitude and pulse-width. While the on-chip digital circuits can be designed to support multiple types of waveforms, this option is constrained by the available chip area, due to high-cost of custom silicon wafer fabrication and utilization of the area by the increasing output-channel count in novel stimulators. Additionally, the set of waveforms remains fixed, and is not adaptable to new stimulation protocols without a major chip redesign.

Figure 3B instead proposes a control architecture distributed among three tiers of devices, all typically present in a miniature neural stimulator system. The system architecture requires the SoC logic to only execute a single stimulation pulse per each data packet received, which relieves the need to define the full stimulation waveform by on-chip circuits. Instead this information is defined and programmed in the external computing device such as a handheld tablet, typically with a graphical interactive User Interface (UI). The UI software takes input from the users or the experiment subjects and generates the necessary, arbitrary-shaped stimulation waveform in a form of a data structure, such as a multidimensional array. The data structure is then transmitted to the Data Relay (DR) module using a ubiquitous link such as Wi-Fi or Bluetooth.

The DR module is located close to the stimulator SoC, either in the same physical enclosure for non-implantable stimulators or just outside of the body for implantable ones. The DR module’s purpose is to establish a reliable transmission (wired or wireless) of stimulation information to the SoC in the form of data packets. Each packet contains parameters for a single stimulation pulse for each channel and a trigger command to “fire” the pulse when received by the SoC. The timing of the packets defines the sampling rate of a continuous arbitrary stimulation waveform, such as in section “Quantifying the Biomimetic Stimulator’s Performance,” or the repetition rate for discrete pulses. As the processing power required to convert a stimulation waveform table to individual data packets is low, the module can be implemented using a low-cost, low-size and low-power Microcontroller Unit (MCU) with a built-in memory. In the implantable configuration, the externally positioned DR is wirelessly linked to the implanted SoC, thus the quality of the wireless link is critical to transmit an uninterrupted stimulation protocol. Reliable wireless connection to the SoC can be readily achieved by one of the integrated data links described in section “Electrical Performance of the Biomimetic Stimulator.”

**FIGURE 4:**
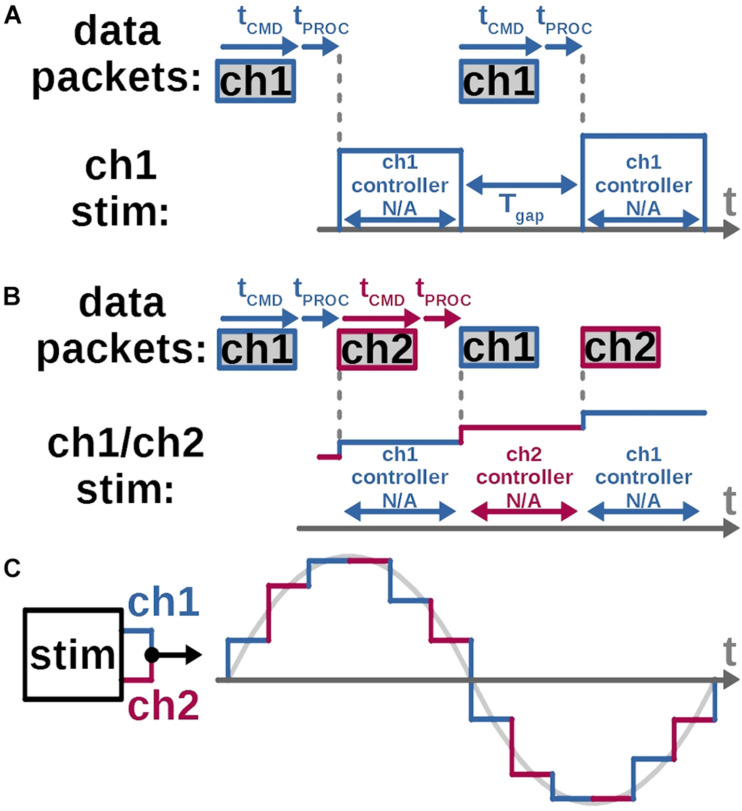
A continuous, gapless arbitrary stimulation waveform is created by interleaving two stimulation channels for each electrode. **(A)** A gap between stimulus pulses arises while using the custom SoC (Lo et al., 2016) adopted for this work, as each channel’s local controller is unable to receive parameters of the next stimulus pulse, while outputting the current one. Thus, SoC ch1’s local controller can accept a new command only after ch1 completes its current output pulse. The resulting gap between current and next pulses equals command length plus command-processing time. **(B)** While ch1 output is in progress, a command is sent and accepted at ch2’s controller, which sets up and fires the next pulse without the gap. **(C)** Arbitrary continuous waveform at each stimulation electrode can be created by connecting it to the output of two channels on the stimulator and assigning odd and even waveform samples to each respective channel.

A unique challenge in the proposed architecture can arise due to limitations of the on-chip digital controller which leaves a small gap in time between any two consecutive output stimulus pulses (Figure 4). This gap may be irrelevant for stimulation protocols with discrete pulse patterns, but it must be carefully managed for continuous biomimetic stimulation waveforms such as in section “Quantifying the Biomimetic Stimulator’s Performance,” where the gaps would add undesired frequency content and thus alter the key characteristics of the waveform. The mitigation of this challenge is illustrated in Figure 4.

In the SoC stimulator employed in this work (Lo et al., 2016) local digital-control circuits for each stimulation channel are individually collocated with the corresponding output current drivers (Kuanfu and Liu, 2009). This enables independent control of each channel’s output timing and amplitude, as demonstrated in section “Bench-Top Test,” and scalability for a higher channel count. But the individual memory register at each channel’s local controller, which holds that channel’s stimulus pulse parameters, does not accept any updates while it’s reading these parameters and outputting the corresponding pulse. The command for the following pulse must be thus sent only after the present pulse is completed (Figure 4A). This creates a minimum gap between each channel’s output pulse equal to the length of the command’s bit stream plus the processing time taken by the local controller. To eliminate the gap, two current output channels can be connected together (Figure 4B). When the first channel is firing an output pulse, a configuration command is sent to and processed by the second channel’s controller, which is set to fire immediately after, without a gap. The gap-less continuous stimulation output with interleaved channels is demonstrated in a bench-top test in section “Bench-Top Test” and the *in vivo* demonstration in section “*In vivo* Tests.”

### Implementation of the Stimulator Prototype

**FIGURE 5:**
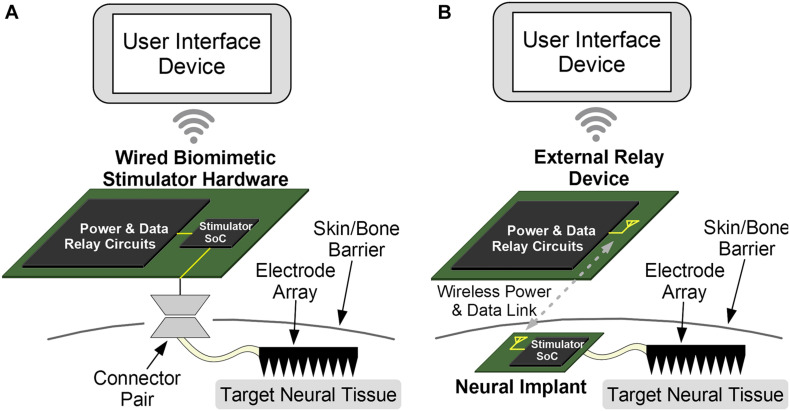
The components of the proposed system can be arranged for wireless or wired implementations. **(A)** While the wired option is easier to implement and provides a stable and constant power, an external connector can disturb normal behavior and has increased risks of infection due to wire-through-skin. This option is best used for prototype testing and acute testing. **(B)** The wireless option is more complex due to challenging wireless data and power links. But the intact skin reduces chances of infection, and chronic experiments can be conducted with normally behaving animals. The wireless approach is further translatable for chronic human therapeutics.

The multi-tier control architecture can be implemented as either a wired or a wireless neural interface. Both options integrate all three components and are shown in Figure 5. The wired arrangement can house the DR circuit on the same substrate, such as a Printed Circuit Board (PCB), as the stimulator SoC and has a physical connection to the stimulating electrode. The wireless implementation instead has an implantable SoC and electrode and usually places the DR module outside of the skin. The UI device can be a detached, handheld device in both cases, making it convenient for the operating user.

While the wired option is easier to implement due to reduced complexity and provides a stable and constant power supply to the SoC, it increases the risk of skin infection due to the protruding physical wire, and is most useful for acute testing of the device or protocols. Even so, the small component-count of this architecture can reduce the size of such “wired” devices and make them portable and convenient for research applications. The wireless configuration requires a wireless data and optionally a wireless power link, although a battery can be implanted as well. This also requires an antenna (for a far-field link) or a coil (for a near-field link). The absence of wires enables chronic applications for therapeutics or testing, assuming a biocompatible packaging for the implanted device. It also allows experiments with freely behaving animals. The Wi-Fi or Bluetooth link between the UI module and the DR module is easily implemented for both options as the two modules are externally positioned and thus are not power or space constrained. The biomimetic stimulator prototype in this work is implemented with the wired option.

**FIGURE 6:**
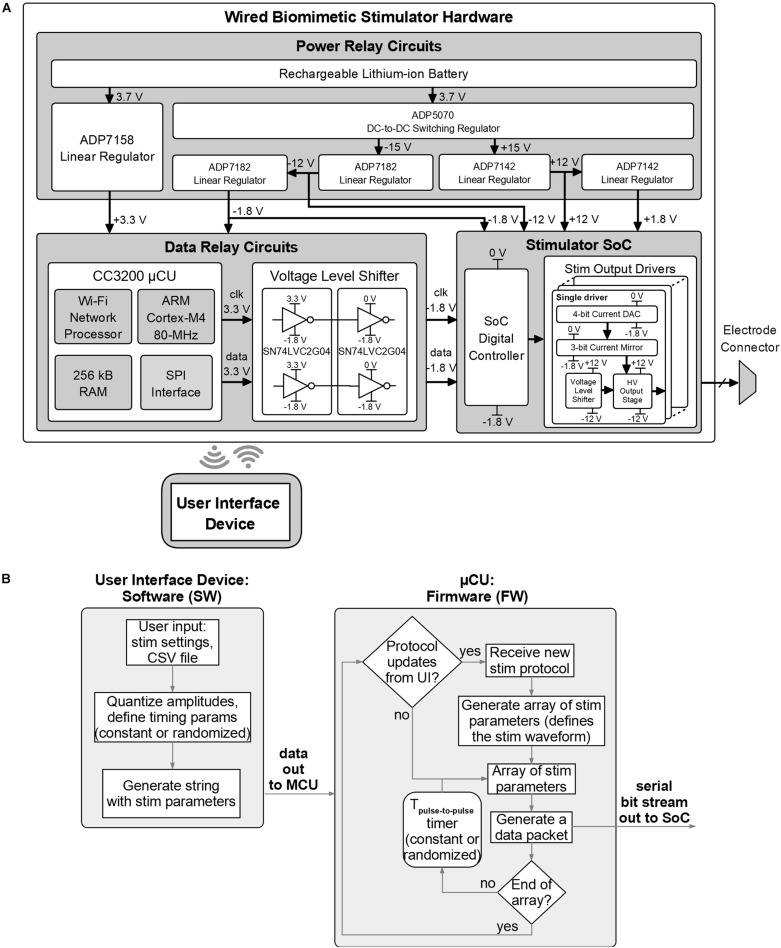
Design details of the biomimetic stimulator prototype. **(A)** Block diagram and schematic of the hardware. Wired arrangement was chosen for initial rapid prototyping and *in vivo* verification. **(B)** Functional blocks of the software and firmware of the prototype.

Figure 6A shows the detailed block diagram of the prototype’s hardware. The hardware is designed in three parts: the power module which provides the required supply voltages, the DR module which receives control from the UI device and sends the necessary command packets to the SoC, and the stimulator SoC whose outputs connect directly to the electrode array. The SoC has an output current range of −500+500 μA per channel with 8-bit resolution (including the sign bit), an output compliance voltage of ±10 V (accounting for headroom required for current sources), can thus accept electrodes with impedance up to ∼20 kΩ at maximum current, and higher for reduced current. The SoC also supports passive charge cancelation to dissipate any residual unbalanced charge during stimulation as needed. These SoC parameters aren’t affected by the control architecture, are directly inherited by the system and recorded in Table 1.

**FIGURE 7:**
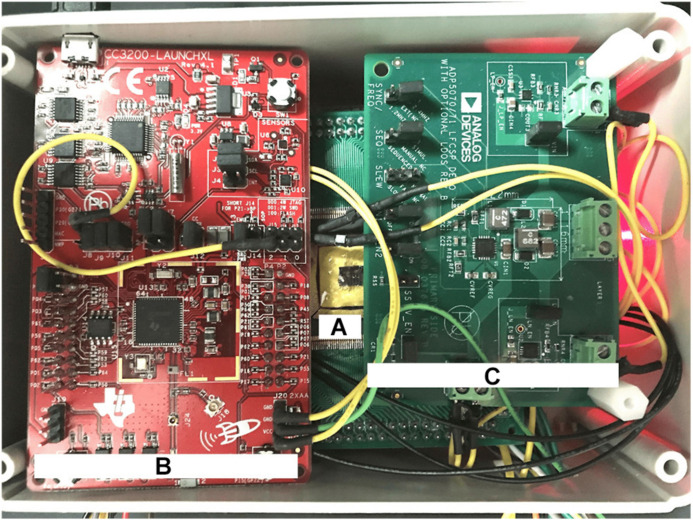
Physical implementation of the wired prototype of the biomimetic stimulator. **(A)** The stimulator SoC in a Quad Flat Package (QFP). **(B)** CC3200 Microcontroller Unit (MCU) from the Data Relay circuits. **(C)** Power relay circuits. Other system components are arranged below the visible ones.

The DR module employs the CC3200 MCU board with custom firmware which takes input from the UI device via Wi-Fi and outputs data via serial to parallel interface (SPI) to the data level shifters. The voltage level shifters translate MCU’s logic levels (0−3.3 V) to levels compatible with the SoC (−1.8–0 V). The power module converts 3.7 V from a rechargeable battery to five regulated supply voltages: ±12 and ±1.8 V for the SoC and 3.3 and −1.8 V for the DR module. The power module uses an off-the-shelf DC-to-DC converter and five low-dropout voltage regulators by Analog Devices (Norwood, MA, United States). The DC-to-DC converter (ADP5070) generates ±15 V from the battery. The ±15 V are then down-converted to ±12 V to supply the SoC by ADP7142 and ADP7182 regulators, respectively. The ±1.8 V for the SoC are then generated from ±12 V through other ADP7142 and ADP7182 regulators. The 3.3 V for the DR circuits is regulated from the battery directly by ADP7158 regulator. Figure 7 shows a photo of the wired prototype hardware which is sized 14 cm× 10 cm× 5.5 cm. The SoC is packaged in a Quad Flat Package (QFP) to interface with the peripheral electronics and the stimulation outputs.

Figure 6B shows the functional diagram of the prototype’s software for the UI device and firmware for the MCU. The software algorithm translates user input, which describes the chosen stimulator protocol, into a sequence of configuration parameters compatible with the stimulator SoC and sends them to the MCU. The MCU generates one data packet on-the-fly for each stimulus pulse in the protocol sequence and sends it to the SoC by the serial interface, following the logic architecture described in section “System Architecture” and Figure 3. The time interval between pulses is controlled by the MCU’s timer and can be a constant or a randomized value, as selected in the user input. The MCU firmware periodically checks for presence of updated protocols from the UI device, to accommodate a closed-loop experiment setup.

### *In vivo* Animal Preparations and Test Procedures

Experiments were performed on two adult rats (250–300 g body weight, one female Sprague Dawley and one male Long Evans). All procedures received approval by the Animal Research Committee of UCLA, and abide by the guidelines provided by the National Institutes of Health (NIH) Guide for the Care and Use of Laboratory Animals. Initially, the animals have been sedated with isoflurane gas, constantly flowing at 1.5−2.5%, followed by urethane (1.2 mg/Kg, i.p). Afterward, the tibialis anterior (TA) and Sol muscles have been bilaterally implanted with recording wire electrodes (AS 632, Cooner Wire Co., Chatsworth, CA, United States) for intramuscular EMG. The recorded EMG signals were band-pass filtered to a 10 Hz to 5 KHz band, notch filtered at 60 Hz, amplified with gain 100 or 1000 using a differential AC amplifier (DP-304A, Warner Instruments, CT, United States) and finally digitized at 20 or 100 KHz (PowerLab^®^, ADInstruments, Australia). Electrical stimulation was delivered using a high-density multi-electrode array fabricated with three longitudinal columns and five horizontal rows of platinum-based electrodes (Chang et al., 2014; Taccola et al., 2020b). The array was implanted in the epidural dorsal space from L1 to S1 spinal levels following a Th12 to L2 vertebrae laminectomy, which dorsally expose the spinal cord. The second rat received a complete transection of the spinal cord performed at Th10 spinal level. To determine the threshold intensity for each preparation, rectangular pulses were delivered at 0.33 Hz starting from 100 μA and increasing at 100 μA increments. The threshold was defined as the minimum intensity which elicited a consistent EMG response from all muscles. Lowering the strength of stimulation to sub-threshold level caused seldom deletions in at least one muscle’s responses. In the intact Sprague Dawley the threshold was equal to 500 μA, was regularly checked during the course of the experiment and remained consistent. To continuously monitor the motor responses, a 0.33 Hz train of test pulses at threshold was delivered before, during and after the application of DS. The effect of DS was quantified in the first 60 s of its application as the change in the peak amplitude of the responses, expressed as a percentage of the respective pre-DS, baseline values. Additionally, animals were kept under anesthesia over a heating pad (37°C) throughout the duration of each experiment. At the end of all experiments (4 h), animals were sacrificed with isoflurane and sodium pentobarbital (IP, 80–100 mg/Kg) followed by a cervical dislocation.

**FIGURE 8:**
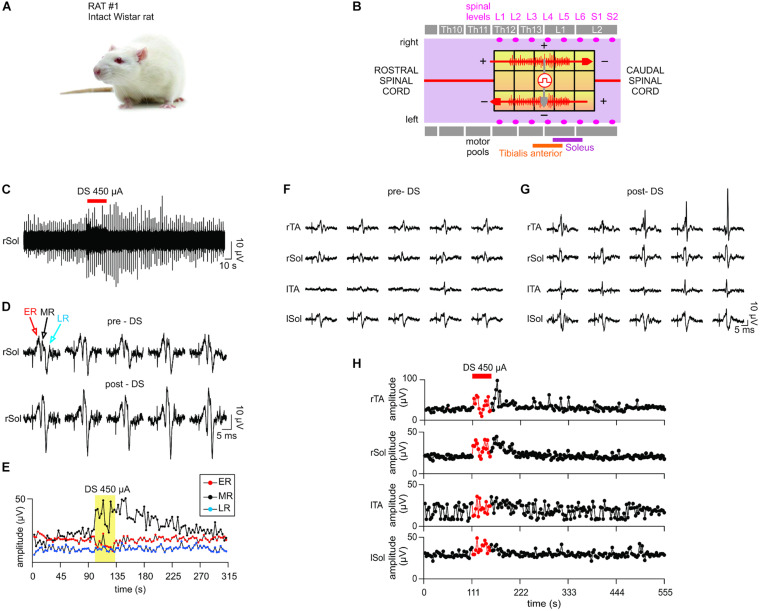
*In vivo* test results I. The stimulator prototype delivers a DS protocol and increases excitability of an intact spinal cord of an animal subject during and post protocol delivery. **(A)** Animal type used in this experiment. **(B)** The diagram of the epidural electrode array shows the electrodes to which stimulation was delivered. Two DS stimuli were delivered simultaneously in a bipolar configuration (red arrows) using the stimulator prototype. An auxiliary stimulation “test pulse” is delivered in the middle of the spinal cord (gray arrow) to test the responsiveness of the motor outputs. The test pulse intensity is adjusted to evoke consistent electromyography (EMG) responses in all muscles. **(C)** DS protocol increases the responsiveness of the motor outputs to test pulses at the right Sol muscle. **(D)** Five evoked response plots were randomly selected from the pre-DS and post-DS recording section to illustrate the resulting range of amplitudes. **(E)** EMG response levels at the right Sol are quantified over time of the experiment. Middle response notably increases due to DS protocol. **(F–H)** The test pulse intensity is reduced to emulate a decreased spinal cord connectivity occurring in cases of spinal cord injury. **(F,G)** While pre-DS responses are relatively decreased or absent, post-DS responses are restored in all muscles. **(H)** Sustained increases of Middle Responses are noted in motor outputs of all four EMG electrodes.

**FIGURE 9:**
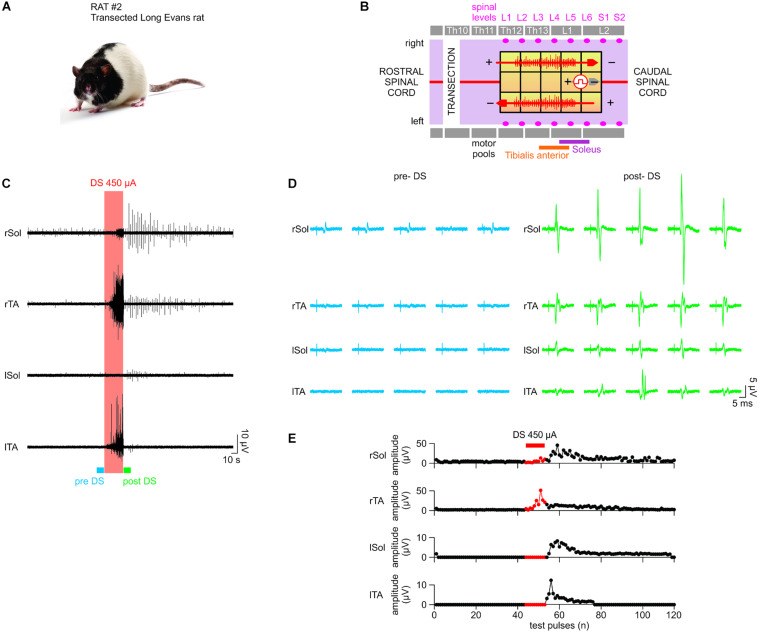
*In vivo* test results II. The stimulator prototype repeats the *in vivo* test in a rat subject with a completely transected spinal cord. **(A)** Animal type used in this experiment. **(B)** Transection is made at the Th10 level (white vertical bar). While the DS protocol is delivered to the same electrode locations as in *in vivo* test I, the test pulse is delivered to different electrodes, which were experimentally chosen to ensure the presence of EMG responses with the spinal cord transection prior to application of DS. **(C–E)** Notably, although consistent responses were not present in the left motor outputs pre-DS, due to the transection, consistent responses appeared on all four EMG electrodes at significantly increased amplitude levels post-DS. This demonstrates the efficacy of the stimulator prototype, which effectively delivers the DS protocol to exploit any spared spinal cord connectivity and restore the motor outputs following a spinal cord injury.

Both the DS and the square test-pulses were delivered concurrently to the same epidural electrode array but at different electrode locations. The prototype stimulator delivered DS to the four corner electrodes of the array, marked by the red arrows in Figures 8B, 9B. As demonstrated previously, this positioning allows this novel protocol and its key properties (section “Quantifying the Biomimetic Stimulator’s Performance”) to increase the recruitment or excitability of the myelinated fibers of the spinal cord under the full area of the electrode array. Simultaneously, a 0.1 ms-wide square pulse repeating at 0.33 Hz was delivered to electrode locations marked with gray arrows (Figures 8B, 9B) by a commercial stimulator STG 4008 (Multi Channel Systems, Reutlingen, Germany). The electrode locations for these square pulses were chosen experimentally in each rat to evoke the EMG responses in the TA and Sol muscles of left and right legs. To ensure consistent EMG responses, the intensity of test-pulses was adjusted as described above and in the section “Results.”

## Results

### Bench-Top Test

**FIGURE 10:**
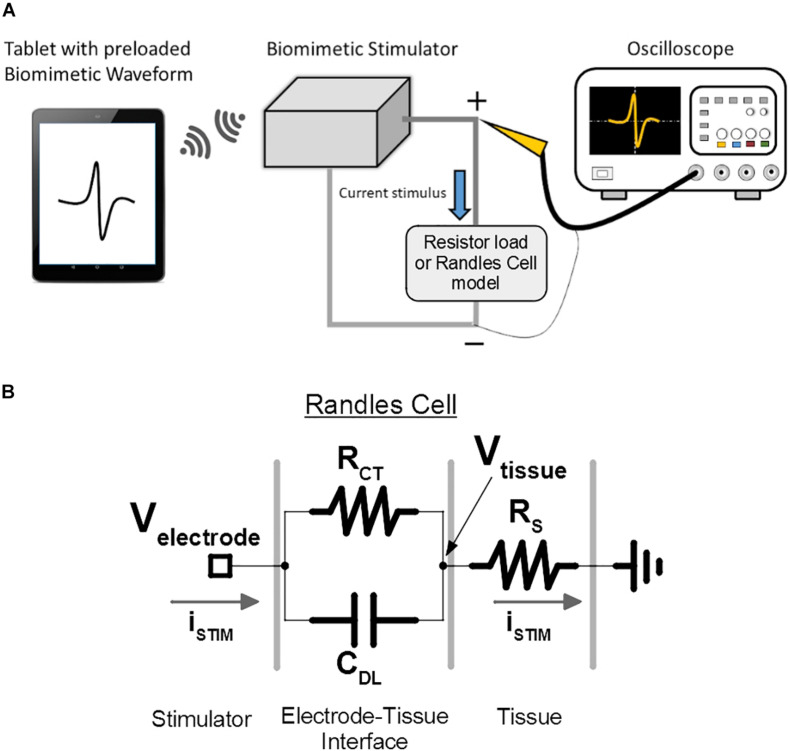
Bench-top test setup is used for verification and demonstration of the prototype of the proposed biomimetic stimulator. **(A)** The illustrated arbitrary waveform is sent to the stimulator, which delivers the current-mode protocol to the emulated loads. The resulting voltage output is captured by the oscilloscope. **(B)** The electrode-tissue interface of the spinal cord epidural electrode is modeled by a Randles cell to investigate the stimulator performance under delivery of DS protocol. V_*tissue*_ node represents the resulting voltage potential experienced by the neural tissue, which doesn’t include the capacitive effects of C_*DL*_. V_*electrode*_ node represents the voltage experienced by the SoC’s output current sources, which includes the effect of C_*DL*_.

The biomimetic stimulator prototype is demonstrated in a bench-top test by generating a variety of waveforms, including a random-period pulse train, a multi-channel arbitrary pulse trains, each with independent parameters, and a continuous biomimetic DS stimulation pattern. The hardware prototype is controlled by the UI app on an Android tablet, which has preloaded test protocols including the biomimetic DS waveform. During the initial functionality demonstration, each stimulation output channel is connected to a 10 kΩ resistor. The oscilloscope captures the resulting waveforms and thus measures the output-current stimuli delivered to the resistive loads (Figure 10A).

Additionally, to better emulate the epidural multi-electrode array used in the *in vivo* experiment during DS protocol output, the resistive load was substituted for a Randles cell circuit (Randles, 1947), which models the electrode-tissue interface (Figure 10B) and its capacitive effects. The C_*DL*_, R_*CT*_, and R_*S*_ values of the model were set to 220 nF, 15 and 1.9 kΩ, as measured during the electrode characterization in previous work.

Importantly, the voltage in a tissue is generally not affected by the R_*CT*_ and C_*DL*_ effects at the electrode interface. This is because the stimulation current will flow into the R_*S*_ of the tissue and out of the ground electrode unaltered by the C_*DL*_, following the Kirchhoff’s laws. Thus, the voltage potential in the tissue, V_*tissue*_, which affects the membrane potentials of the targeted neurons, will be created by the stimulation current and only R_*S*_ (reduced in magnitude depending on how far the neuron is located from the site of stimulation). The stimulator’s performance is then validated using the recorded V_*tissue*_ signal. Still, for the above to be valid, V_*electrode*_ potential must not exceed the compliance voltage of the SoC current sources, otherwise the output current will be distorted from the intended values. This is validated by analysis of V_*electrode*_ signal recorded during the DS output.

**FIGURE 11:**
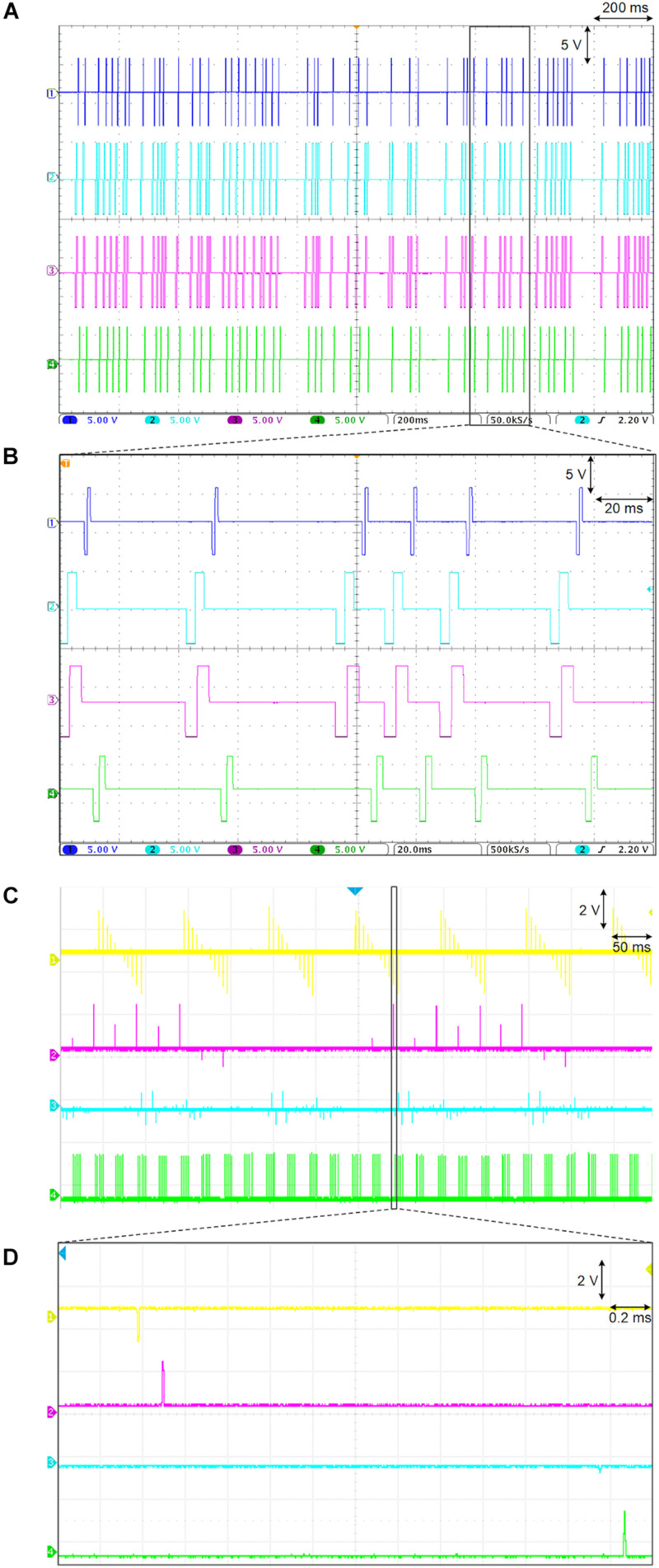
Bench-top test results demonstrate a variety of pulse trains protocols. **(A)** Multi-channel stimulation with random inter-pulse-interval, which follows an exponential distribution. **(B)** Magnified view of panel **(A)** shows the different pulse widths and start delays among different channels. **(C)** Multi-channel stimulation with independent control of waveform, frequency, amplitude and pulse timing. **(D)** Magnified view of panel **(C)**.

The randomized period pulse train stimulation is demonstrated in Figures 11A,B The resulting multi-channel pulse trains exhibit random IPI which follows an exponential random distribution and has been shown to reduce undesired neural adaptation in epiretinal stimulation (Soto-Breceda et al., 2018). The mean period is 30 ms with current amplitudes set to 0.5 mA and pulse width set between 1 and 4 ms among the available channels. Pulse timing offset (phase) can be set among the stimulation channels during this random IPI protocol, which further demonstrates versatility of the prototype’s logic architecture. Figures 11C,D demonstrates the capability to output multi-channel arbitrary pulse trains, each with independent control of waveform, frequency, and amplitude. This protocol was output simultaneously on 16 channels but only the first four channels are shown due to the limitation of the four-channel oscilloscope. The oscilloscope’s channel 1 (yellow) demonstrates a ramp waveform on both anodic and cathodic phases at 200 Hz with a 10 Hz pulse train frequency. Channels 2 (pink) and 3 (blue) demonstrate random firing patterns at low and high frequency (40, 250 Hz). Channel 4 (green) demonstrates a high frequency burst (500 Hz) with a burst-repetition frequency of 40 Hz.

**FIGURE 12:**
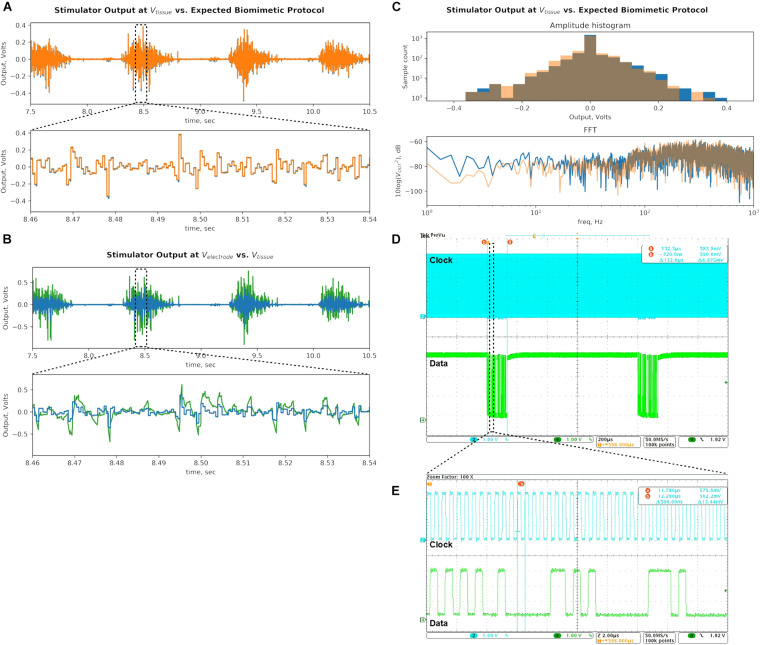
Bench-top demonstration of multi-channel continuous DS waveform mimics a recorded EMG signal, which has been shown to be effective in epidural spinal cord stimulation for restoring motor function. **(A)** Stimulator output recorded at V_*tissue*_ (blue) is compared to the desired biomimetic protocol waveform (orange). The signals are similar as intended, demonstrating the prototype’s ability to deliver gapless and continuous stimulation protocols. **(B)** Output recorded at V_*electrode*_ (green) is compared to the signal at V_*tissue*_ (blue). The electrode signal is higher in amplitude due to the added voltage drop across the C_*DL*_ capacitor, which integrates the output current pulses, but is within the compliance limits of the SoC output current sources with significant margin. **(C)** The DS output of the stimulator (blue) is analyzed against the desired biomimetic protocol (orange). The biomimetic current protocol is multiplied by the R_*S*_ value to convert it to voltage units. Histograms of amplitudes of signal peaks and frequency spectrums of the signals are plotted. **(D)** Clock and data digital inputs to the SoC are plotted. Spacing between data packets identifies the sampling rate of the output stimulus signal. Total time period of a packet helps calculate the number of bits it contains, by using the clock period **(E)**. Combination of information in panels **(D,E)** yields the total data bitrate of the DS biomimetic stimulation waveform.

Figure 12 demonstrates the prototype’s ability to generate a continuous biomimetic stimulation waveform. The signal is preprocessed as in section “Quantifying the Biomimetic Stimulator’s Performance” and loaded into the UI device. The UI device’s software further interleaves the signal onto two SoC outputs for gapless waveform. Figure 12A shows the resulting DS output (blue) generated by the stimulator as measured at V_*tissue*_ Randles cell node. The desired DS current-mode protocol (orange) from section “Quantifying the Biomimetic Stimulator’s Performance” is then multiplied by the R_*S*_ value to convert the data to voltage and to match the scale of the recorded signal. The zoomed in bottom panel shows that the two waveforms are similar, aside from the high-frequency noise aliased into the signal band due to low sampling frequency of the oscilloscope when recording long signals, and its lack of anti-aliasing filter. The signal is quantized as expected and the temporal resolution of 500 μs preserves the original signal’s sampling rate of 2 ksps.

Next, Figure 12B compares the signal recorded at V_*electrode*_ (green) against the signal at V_*tissue*_ (blue). The V_*electrode*_ signal is larger in amplitude because the R_*CT*_ and C_*DL*_ voltage drop is in series with the R_*S*_ voltage. The minimum and maximum potentials at the V_*electrode*_ during DS output are measured to be −0.903 and 0.760 V, respectively. These values are much lower than the ±10 V compliance limits of the SoC current sources, facilitating them to deliver the intended output current. The bottom panel zooms in on the V_*electrode*_ waveform, which exhibits RC exponential settling as the current pulses are integrated by the C_*DL*_, which is also superimposed on the V_*tissue*_ potential. The charge build-up on the capacitor is frequently discharged when the DS signal changes polarity. This DS protocol output delivered by the stimulator prototype was tested *in vivo* as described in section “*In vivo* Tests.”

Following the methods of section “Signal Analysis of the Biomimetic Protocol,” the histogram of peak amplitudes, and the frequency spectrum of the signal are plotted in Figure 12C (blue). The desired DS signal multiplied by R_*S*_ (as above) is also analyzed and superimposed on the plot (orange). The comparison of the two signals demonstrate that the modulations in amplitude and frequency at the output are largely preserved as intended. Still, a few differences are noted.

The FFT of the prototype’s output has higher content in the 60-Hz band due to 60 Hz noise present on the bench top setup. Low frequency band exhibits higher power, possibly due to aliased high-frequency switching noise as explained above. The same added noise likely affected the output signal’s histogram (blue) by pushing some of the peaks from their original bins into the neighboring ones. It’s noticeable in the −0.250 V bin, where the peak count was low to begin with.

Quantitatively, the range of the amplitude peaks of the output signal are 0.376 and 0.418 V in positive and negative directions, respectively. These values deviate by +9.3% and −2.3% from the corresponding values of the original DS signal. The Wiener entropy for the captured output is −1.87 dB, which deviates by +0.32 dB or 3.8% on a linear scale. The measured values have deviated from the ideal by less than 10%, mostly in the direction of higher levels of modulation in amplitude and frequency. The values are recorded in Table 1, and the efficacy of the resulting output signal with respect to the therapeutic effect is empirically verified *in vivo* as described in the next section.

Finally, the command data rate is verified by capturing the data and clock signals at the SoC digital inputs (Figure 12D). The number of bits in a data packet can be calculated by measuring the distance between the start and end of the packet (133 μs) and dividing it by the clock period (here: 0.5 μs, Figure 12E). Each resulting 266-bit command packet produces two consecutive pulses in an output with 0.5 ms width each (per the gapless schema in section “Logic Architecture for Arbitrary Waveform Generation”). This command repeats with a period of 1 ms. The total data bitrate is 266 bits/packet × 1000 packets/sec = 133 kbps. The value is recorded in the Table 1 and is well within the bitrate supported by the data links discussed in sections “Quantifying the Biomimetic Stimulator’s Performance” and “Simultaneous Power and Data Telemetry in an Implantable Implementation.”

### *In vivo* Tests

The efficacy of the proposed architecture was demonstrated *in vivo* with DS protocol. The *in vivo* test is a shorter version of the previously published *in vivo* works with this protocol. As shown, DS delivered by the prototyped device is able to increase the excitability of intact and transected spinal cords in rats. The excitability was concurrently measured by administering a series of current-mode square test-pulses at the spinal cord and recording the amplitude of the EMG responses from leg muscles. The details of the *in vivo* results are described below.

#### *In vivo* Test I Results: Intact Spinal Cord

A Sprague Dawley rat with the intact spinal cord (Figure 8A) exhibited consistent motor responses in all four EMG electrodes for 0.1-ms test-pulses at threshold amplitude of 500 μA administered to L3/L4 levels (Figure 8B). EMG responses from right Sol muscle before, during and after DS protocol, are shown in Figure 8C. Zoom-in plots in Figure 8D show distinct early response (ER), middle response (MR) and late response (LR) (Gerasimenko et al., 2008) with different amplitudes pre-DS as compared to post-DS. Pre-DS peak values are: ER = 17.98 ± 1.35 μV, MR = 22.67 ± 2.59 μV and LR = 10.06 ± 1.78 μV, averaged for *n* = 20 repeated pulses, as quantified in Figure 8E. Based on the same plot, the change in EMG responses due to the delivery of DS protocol can be calculated as percentage of pre-DS levels. The MRs increased to 167%, while ERs and LRs changed to 77 and 102%, respectively, as averaged for *n* = 10. The effect of DS on the amplitude of MRs persisted for at least 1 min after its end, peaking at 170%, averaged for *n* = 20.

The experiment was repeated with test pulses at a reduced, sub-threshold level of 350 μA, while DS was kept at the same level. This test emulates the reduced signaling condition which occurs when an injury to the spinal cord decreases its connectivity. Due to the sub-threshold level of test pulses, pre-DS MRs were minimal (Figure 8F) and inconsistent, with noted deletions. Again, the DS protocol increased the responses in all muscles (Figure 8G). Average pre-DS MR levels were measured as 27.56 ± 4.01 μV for rTA; 20.52 ± 1.51 μV for rSol; 17.22 ± 8.38 μV for lTA and 29.12 ± 3.52 μV for lSol at *n* = 20 (Figure 8H). During DS the levels increased to rTA = 134%; rSol = 148%; lTA = 124%; lSol = 126% of pre-DS with *n* = 10. The increase has persisted post-DS, peaking at rTA = 164%; rSol = 153%; lTA = 148%; lSol = 113% of pre-DS levels, with *n* = 20.

#### *In vivo* Test II Results: Transected Spinal Cord

A Long Evans male rat with a transected spinal cord (Figure 9A) was used as the test subject. Unlike in the first rat experiment with intact spinal cord, no intensity of test-pulses, including maximum available 800 μA, was able to produce consistent EMG responses on all four leg muscles pre-DS administration due to spinal cord transection, which intentionally damaged the neural connectivity. Instead, 0.1 ms, 0.33 Hz, and 650 μA test-pulses were delivered to the epidural electrode array (Figure 9B), which consistently evoked EMG responses on the right Sol and TA muscles only. It is desired to restore the responses on the left muscles by increasing the spinal cord excitability post-transection using the prototype system with DS protocol (Figure 9C). In Figure 9D, left, five sample pre-DS responses are shown with average (*n* = 5) response levels of 3.97 ± 1.02 μV for right Sol and 1.81 ± 2.37 μV for right TA. The asymmetry in the response levels and consistencies among left and right side is most likely due to the transection which unequally affects each specific motor pool.

During DS, responses continued on the right side, but also appeared on the left TA (Figure 9C, red box). Post-DS average (*n* = 5) response levels on the right side increased to 17.41 ± 10.08 μV for rSol and 11.81 ± 3.39 μV for rTA (Figure 9D, right). Also, post-DS responses appeared on the left side with average (*n* = 5) levels of 5.91 ± 2.49 μV for left Sol and 5.97 ± 3.75 μV for left TA. Figure 9E shows the EMG peak levels over time. During DS response levels on the right side increased to 127% for right Sol and 896% right TA relative to pre-DS, and remained increased post-DS at 520% right Sol, 599% right TA for up to 1 min. Most notably, the left TA responses, absent pre- and during DS were noticeable post-DS for up to 1 min at 3.06 ± 2.39 μV for *n* = 23. Also left Sol responses appeared and persisted post-DS for 3 min at 2.67 ± 1.77 μV for *n* = 59. The test data strongly suggests that the administration of DS, rendered by the prototype system, has successfully increased excitability of the spinal cord and thereby effectively re-established its connectivity after the complete transection.

## Discussion

In addition to implantable applications, the prototype can also be advanced to provide biomimetic or other versatile stimulation waveforms in closed-loop neuromodulation. To that end this section includes the relevant design considerations for the wireless link between DR and SoC components and an additional neural recording component.

### Comparison to State of the Art

Table 2 compares the proposed architecture and its prototype with other state of the art. To the best of the authors’ knowledge, this work is the first *in vivo* demonstration of an SoC-based stimulator architecture which supports a continuous, gapless biomimetic waveform and is fully compatible with integration into implantable neural prosthetics.

**TABLE 2:** Comparison of state-of-the-art SoC-based arbitrary waveform neural stimulators.

State of art	I_*OUT*_ max	I_*OUT*_ resolution, bits	# of stim chan	Continuous gapless stim?	Max. length of arbitrary waveform, (# points)	Demonstrated *in vivo*?
Piech et al. (2020)	400 μA	3-bits	1	No	Not SoC limited^1^	Yes
Yip et al. (2015)	500 μA	6-bits	8	No	8/phase	No
Kassiri et al. (2017)	1.35 mA	8-bits	64	No	32/phase	Yes
Noorsal et al. (2012)	1 mA	5-bits^2^	1024	No	64	Yes
This work	500 μA	8-bits	32	Yes	Not SoC limited^1^	Yes

*^1^Waveform is stored outside of the SoC in an external device and is limited by the PROM size, which can be chosen arbitrarily large with respect to the length of known biomimetic waveforms. ^2^Design provides limited control of the shape of the 64-point arbitrary waveform within the I_OUT_ resolution. E.g., each subsequent point can be increased or decreased by 1 LSB relative to the previous or scaled by 0.5× or 2× relative to previous, but the absolute value of each point can’t be set.*

### Simultaneous Power and Data Telemetry in an Implantable Implementation

In an implantable implementation, the proposed control architecture requires the SoC component to continuously receive data packets containing the biomimetic stimulation protocol, while simultaneously receiving wireless power. The SoC used in this system employs dedicated circuits on-chip to receive data and power transmission using a near-field, inductive coupling. In an implant, the SoC is connected to a pair of coils which receive the data carrier signal at 20 MHz and the inductive power signal at 2 MHz. The 10× difference in frequencies allows on-chip filters to separate the data signal from the 2 MHz interference. The data is transmitted at a rate of 2 Mbps with DPSK modulation and is decoded by the SoC into packets for the digital controller. On-chip quad-level rectifier and regulators convert the power signal into four supply voltages required for SoC operation.

This wireless schema was previously demonstrated in a spinal implant, as well in a high-density epiretinal prosthesis (Lo et al., 2013). Importantly, the data rate of 2 Mbps enabled the epiretinal implant to receive a 1024-pixel (i.e., stimulator channels) image, continuously refreshed at 60 frames per second, with each pixel sample requiring a 19-bit packet, under wireless power. This resulted in a data rate of 1.17 Mbps plus overhead, which is significantly higher than the one measured for the proposed biomimetic stimulator, as indicated in Table 1, and thus meets the needs of the implantable implementation.

### Stimulation Safety Mechanisms

Importantly, the proposed control logic, which distributes the control over three components of the system, does not increase the risk of delivering an unsafe amount of charge during stimulation. Moreover, three safety mechanisms are built into the proposed system, to further mitigate this risk.

First, the biomimetic signal is chosen to have a zero DC component. This is achieved by recording the EMG (DS) signal with a high pass filter, thus eliminating any arithmetic mean component and resulting in net-zero charge during stimulation. Second, every command sent to the SoC includes one bit which can turn on an optional passive charge dissipation at the electrode. The MCU can periodically enable it at predefined time intervals dissipated any net charge during the stimulation. Third, if any command between UI Device and MCU, or between MCU and SoC is corrupted, the built-in error check mechanism would discard the command. This check is a part of the Wifi standard, and is included as CRC and checksum in our SoC command structure. If the corrupted command is discarded then no corresponding stimulation pulse will occur, thus safely underdelivering the stimulus charge. No reasonable chance exists for a corrupted command to be accepted and erroneously overdeliver an unsafe, large amount of charge.

### Stimulation Artifacts in Neural Recordings During Continuous Biomimetic Stimulation

To gain new insights into the mechanisms and efficacy of novel biomimetic stimulation protocols, it can be advantageous to record neural activity concurrently with stimulation to help investigate the neural network’s dynamics under these protocols. For example, DS protocol is speculated to increase the excitability of spinal networks and specifically the recruitment of spinal cord’s interneurons to generate a more robust motor response. Further evidence may be acquired by monitoring the cord’s neural signals before, during and after DS stimulus. Yet, the stimulation signal injected into the electrode-tissue interface creates an undesired stimulation artifact that is recorded alongside the neural activity. The artifact is frequently larger than the neural signal of interest and can confound the latter (Hottowy et al., 2012). Although solutions to artifact removal have been developed for protocols with periodic stimulation pulses (Stanslaski et al., 2012; Mendrela et al., 2016; Basir-Kazeruni et al., 2017; Culaclii et al., 2018; Zhou et al., 2019), removal of artifacts from continuous complex stimulation waveforms, such as DS, are yet to be demonstrated. Still, a system level approach in Culaclii et al. (2018), which learns the initial artifact template and subsequently subtracts it from the recurring artifacts in the recordings, can be extended to accommodate the continuous artifacts from DS. This approach uses an MCU and data converters which interact with the recording amplifiers to perform the learning and removal. The MCU’s memory can be increased as needed to store the continuous artifact spanning the full duration of the DS protocol.

## Conclusion

A novel architecture is proposed for next generation neural stimulators to support a multitude of irregular, non-tonic stimulation waveforms, simultaneous multi-frequency output on multiple channels, and most notably continuous, gap-less arbitrary biomimetic waveforms from pre-recorded physiological signals. In contrast to the conventional approach which places the waveform generation onto the SoC component only, the proposed architecture additionally integrates firmware and software components and distributes the waveform generating logic over all three resulting domains in the stimulator system. The proposed approach is fully compatible with a design of a neural implant. A portable stimulator prototype is built and tested at bench-top to demonstrate the supported waveforms. The stimulator is also used *in vivo* in animal experiments, where it successfully delivers a biomimetic waveform to exploit the spared connectivity along a transected spinal cord and to restore the motor output in a rat model. The integration of the proposed system as a neural implant and its *in vivo* demonstration in freely behaving animals is planned for future work.

## Data Availability Statement

The raw data supporting the conclusions of this article will be made available by the authors, without undue reservation.

## Ethics Statement

The animal study was reviewed and approved by Animal Research Committee of UCLA.

## Author Contributions

WL and SC: biomimetic stimulator concept and system architecture. SC, P-MW, and Y-KL: circuit design. WY and BB: firmware and software development. Y-PC and SC: stimulation signal analysis. GT, SC, WY, and WL: *in vivo* testing. GT: physiological data interpretation. SC, P-MW, GT, WL, and Y-KL: write the manuscript. All authors contributed to the article and approved the submitted version.

## Conflict of Interest

WL and Y-KL hold shareholder interest in Niche Biomedical Inc. P-MW was employed by the company Niche Biomedical Inc. The remaining authors declare that the research was conducted in the absence of any commercial or financial relationships that could be construed as a potential conflict of interest.

## Publisher’s Note

All claims expressed in this article are solely those of the authors and do not necessarily represent those of their affiliated organizations, or those of the publisher, the editors and the reviewers. Any product that may be evaluated in this article, or claim that may be made by its manufacturer, is not guaranteed or endorsed by the publisher.
